# Towards comprehensive early abortion service delivery in high income countries: insights for improving universal access to abortion in Australia

**DOI:** 10.1186/s12913-016-1846-z

**Published:** 2016-10-22

**Authors:** Angela Dawson, Deborah Bateson, Jane Estoesta, Elizabeth Sullivan

**Affiliations:** 1 Faculty of Health, University of Technology, Sydney (UTS), P.O. Box 123, Ultimo, NSW 2007, Sydney, NSW Australia; 2 Discipline, Gynaecology and Neonatology, University of Sydney, Family Planning New South Wales, 28-336 Liverpool Road, Ashfield, NSW 2131 Australia; 3 Family Planning New South Wales, 28-336 Liverpool Road, Ashfield, NSW 2131 Australia; 4 Public Health, Faculty of Health, University of Technology, Sydney (UTS), Jones Street, Sydney, NSW Australia

## Abstract

**Background:**

Improving access to safe abortion is an essential strategy in the provision of universal access to reproductive health care. Australians are largely supportive of the provision of abortion and its decriminalization. However, the lack of data and the complex legal and service delivery situation impacts upon access for women seeking an early termination of pregnancy. There are no systematic reviews from a health services perspective to help direct health planners and policy makers to improve access comprehensive medical and early surgical abortion in high income countries. This review therefore aims to identify quality studies of abortion services to provide insight into how access to services can be improved in Australia.

**Methods:**

We undertook a structured search of six bibliographic databases and hand-searching to ascertain peer reviewed primary research in English between 2005 and 2015. Qualitative and quantitative study designs were deemed suitable for inclusion. A deductive content analysis methodology was employed to analyse selected manuscripts based upon a framework we developed to examine access to early abortion services.

**Results:**

This review identified the dimensions of access to surgical and medical abortion at clinic or hospital-outpatient based abortion services, as well as new service delivery approaches utilising a remote telemedicine approach. A range of factors, mostly from studies in the United Kingdom and United States of America were found to facilitate improved access to abortion, in particular, flexible service delivery approaches that provide women with cost effective options and technology based services. Standards, recommendations and targets were also identified that provided services and providers with guidance regarding the quality of abortion care.

**Conclusions:**

Key insights for service delivery in Australia include the: establishment of standards, provision of choice of procedure, improved provider education and training and the expansion of telemedicine for medical abortion. However, to implement such directives leadership is required from Australian medical, nursing, midwifery and pharmacy practitioners, academic faculties and their associated professional associations. In addition, political will is needed to nationally decriminalise abortion and ensure dedicated public provision that is based on comprehensive models tailored for all populations.

## Background

Access to a safe, early induced abortion, or the termination of an unplanned pregnancy (up to and including 13 weeks [1] can potentially prevent the death of nearly half a million women and the associated morbidity of another five million women worldwide [2]. Improving access to safe abortion is an essential strategy in the provision of universal access to reproductive health and achieving the sustainable development goals [3].

Comprehensive safe abortion care encompasses the provision of elective abortion services at the request of the woman, along with counselling for contraceptive use, medical after-care, and attention to other issues that are relevant to the woman’s health [4]. The provision of safe medical and/or surgical abortion requires an enabling legal, regulatory and policy environment that is responsive to women’s needs and community demands for quality care, services and information. The legal grounds upon which a woman is permitted to have an abortion differ across and within countries. However, a woman’s access can also be affected by the: availability and quality of health services, geographical access to services, financial affordability and socio-cultural acceptability of the procedure/s and associated care.

Planning, delivering and evaluating legal and safe, comprehensive abortion services that enable women to make a choice about the use of such services requires attention to a number of principles that were developed by the World Health Organization (WHO) through a high level consultative process. These quality service delivery principles include the establishment of national standards and guidelines, integrated multidisciplinary services, ensuring health-care provider skills and performance, the costing and financing of services, and a systematic approach to policy and programme development.

Despite numerous guidelines and recommendations there has been little research from a health services approach to help direct health planners and policy makers to increase access to abortion. Souza et al. have noted gaps in best practice with regard to integrating abortion services into existing family planning services and optimizing the workforce [5]. However, although task shifting from doctors to nurses and midwives in high income countries (HIC) (countries with a gross national income per capita of $12,476 or greater) [6] shows promise [7], there is a lack of evidence in this area [8]. The few systematic reviews that are available in the area of service delivery include an examination of the acceptability of home and clinic based medical abortion by Ngo et al. [9] in largely low and middle income countries (LMIC) with the exception of one study from France. Another review comprising three studies from Italy, Scotland and Iceland found no evidence of increased acceptance and use of contraceptive methods after an abortion [10]. The review by Doren at al. [11] examines factors that facilitate or constrain access to abortion from the perspective of women and providers. Despite these studies there are no systematic reviews that provide insight into models of comprehensive medical and surgical abortion services. This review therefore aims to identify quality studies of abortion services to provide insight into how access to services can be improved in Australia.

### The Australian context

There is no national data collection on the incidence of induced abortion in Australia. Notification of abortions is mandatory in the States of South Australia, Western Australia and the Norther Territory but only South Australia and Western Australia publish data on induced abortions [12, 13]. Several studies have estimated the number of abortions in Australia of over 80,000. The estimated number of induced abortions adjusted for patients who do not claim Medicare (National Reimbursement Services) were 84218 and 83210 in 2013 and 2014, respectively. The national age-standardised abortion rate was 19.7 per 1000 women in 2003 and 19.3 per 1000 women in 2004. However, the latest information was from 2004 [14–16].

The situation in Australia is further complicated with marked differences in abortion legislation between states and territories. Of the nine laws that govern abortion in the country, restrictions exist in all with the exception of the Australian Capital Territory. This highlights the urgent need for reform [17]. The recent focus at the national level has been on new Medicare Benefit payments for pregnancy support counselling that has been poorly accessed by women with unplanned pregnancies [18].

The Australian public including health professionals are largely supportive of the provision of abortion [19, 20] and its decriminalization [21]. However, the lack of data and the complex legal and service delivery situation impacts upon access for women seeking an early termination of pregnancy. Recent literature cites issues related to geographical and financial access [22, 23] and considerable gaps in women’s knowledge [24]. Services are provided across a myriad of public and private contexts where there is no accepted and standardized approach for service delivery that facilitates universal access to comprehensive abortion care. This paper therefore aims to identify service delivery models of comprehensive abortion care, both medical and surgical, that has been effective in increasing access to early abortion care in high income country settings. Such insights may provide Australia with practical evidence-based policy options for improving access to abortion services.

### A framework for understanding the factors contributing to universal access to comprehensive abortion services

Access can be understood from supply and demand side perspectives [25]. Demand for a service is influenced by factors that determine whether a woman is willing and able to seek appropriate abortion care. Supply is determined by factors that include availability, technology, management, and price that interact to produce effective health care. These perspectives can be considered under various dimensions of access that are well described in the literature [26, 27]. We have summarised these dimensions in a framework adapted from existing literature [28, 29] (see Table 1) in order to conceptualise questions that can be used to examine the extent of access to abortion services.

**Table 1:** A framework for examining access to early abortion services

Access Dimensions	Questions to establish extent of access to comprehensive abortion services-what is done and how
*Availability*: The number of existing abortion services meet women’s needs	How many and what types of abortion services exist? Which organizations offer these services? Are there enough willing and skilled personnel to deliver abortion services? Do the services offer choices that correspond with the needs of financially disadvantaged women? Are the services and supplies of abortion drugs and associated equipment and tests sufficient to cover the demand?
*Accessibility*: Abortion services are near where women live, or accessible in their homes and referral timely	What is the geographical distance between abortion services and the homes of women? Can services be reached and women referred in a timely and stress-free manner? How has technology been employed to address issues of distance?
*Affordability*: The prices of terminations are aligned with women’s income and ability to pay	What are the direct costs of abortion services and the associated commodities? What are the indirect costs in terms of transportation, childcare, lost time and income? Are payment options available for women? What are the costs to the health system and are they affordable?
*Adequacy*: The organization of abortion services meet women’s expectations	How are/is the abortion service/s organized? Does the organizational structure meet women’s expectations? Do opening hours match the schedules of women? Are facilities clean, organized and well managed?
*Acceptability*: The services are satisfactory and used by women	Does the information, explanation, and service provided accommodate social norms and values? Do women find self-management aspects such as drug taking and pregnancy testing satisfactory and easy to use? Do women feel welcome and cared for? Do women trust in the competence and character of the health care providers?
*Quality*: The services are scientifically and medically appropriate and of good quality	Are there service delivery standards and quality management systems? How are women’s complaints dealt with? What quality assurance, control and improvement mechanisms are in place? Quality also extends to the manner in which women are treated, and how underlying determinants of health are addressed. Is there provider sensitivity training (i.e. in appropriate language use, cultural safety, privacy) or/and incentive schemes for providers to offer services? Are providers accredited, and regulated? Do providers collect accurate data? Is family planning/contraceptive advice provided? How are abortifacient drugs packaged? Is treatment observed if required? What family planning training and peer support is available for providers?

## Methods

Six bibliographic databases (Medline, PubMed, Web of Science, ProQuest Health and Medicine, Scopus, Science Direct), Google Scholar and the reference lists of identified papers were methodically searched to retrieve research literature. A deductive qualitative content analysis methodology was employed to analyse selected primary research manuscripts [30].

A Population, Interventions, Comparators, Outcomes, Study design question guided the development of the review question [31]. The question we sought to answer was: for services providing first trimester abortion in high income country settings, what approaches to service delivery have been found to be effective in increasing access? We aimed to include studies with demonstrable outcomes from both supply and demand perspectives while acknowledging important health systems factors that enable service delivery. We sought studies examining comprehensive abortion services, as well those investigating components of abortion services with a focus on understanding new or existing delivery systems and key processes as defined by Donadedian [32]. Outcomes of interests include the abortion service delivered, changes in behaviour such as contraception uptake, change in health status such as complete terminations and women’s access to abortions services as defined in Table 1 including satisfaction and uptake of services.

We defined comprehensive abortion service delivery as the provision of legal, safe, stigma free, high quality services that include abortion, post abortion care, contraception and referral. This also involves attention to issues concerning information provision, initial assessment and arrangements for the procedure as outlined in the recent best practice paper by the Royal College of Obstetricians and Gynaecologists (RCOG) [33] and service factors identified by IPAS [34].

**Fig. 1:**
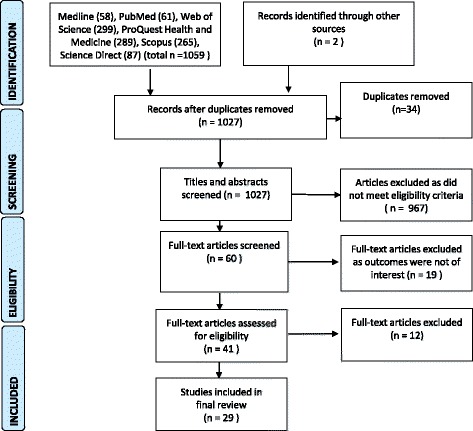
The Literature review selection process. Preferred Reporting diagram for Systematic Reviews and Meta-analyses (PRISMA) showing selection of publications for review

In line with other systematic reviews in the field [35, 36] a structured search of contemporary research literature was conducted between 2005 and 2015 using key words: “abortion” OR “termination of pregnancy” AND “service delivery” OR “model of care”. We sought to assess the results of peer reviewed primary research literature therefore grey resources were excluded. Retrieved records were first screened for their focus on abortion service delivery according to the review question by the first author and duplicates removed. According to the inclusion/exclusion criteria (see Table 2) studies whose focus was outside of the aim, along with discursive papers and those older than 10 years were removed. The Preferred Reporting Items for Systematic Reviews and Meta-Analyses (PRISMA) guidelines were employed to report the review process [37] (see Fig. 1). The sources and numbers of papers retrieved and screened according to their relevance are outlined at Table 3. Most papers were then excluded (999) at closer examination as they were not concerned with specific service delivery aspects the paper by Loeber is one example [38].

**Table 2:** The inclusion/exclusion criteria applied to the screening of papers for the review

*Included*	*Excluded*
Health service research examining how women access health providers and early abortion services, how much care costs, and what happens to women as a result of this care	Clinical studies examining safety and effectiveness of medications, devices, diagnostic products and treatment regimens intended for early abortion
Research examining first trimester abortion service delivery processes, activities, strategies or components with the goal of improving provision, quality, utilization, coverage, efficiency, and equity	Research examining input aspects or resources of abortion care such as human resources, insurance schemes, drug supply or procurement, national policy or governance
Studies that include women’s and provider’s experiences of an actual early abortion service or specific aspects of the delivery of the service or approaches involved	Studies exploring women’s preferences, reasons, opinions for or about services if they needed them. Papers concerning health professional’s general experiences of provision or interest in provision
Primary research paper	Discursive or descriptive outlines of projects, conference abstracts
High income country	Upper middle, middle, lower middle and low income country setting
English	Non English
>2005	<2005

**Table 3:** Summary of all papers included in the review

Reference	Context	Method	Sample/participants	Aim	Findings
(Astle, Cameron & Johnstone 2012) [54]	Medical abortion: National Health Service hospital Scotland UK	Quantitative descriptive: A retrospective audit of facility data	1128 women undergoing medical abortion (up to 64 days’ gestation) over 9-month period	To establish how early medical discharge impacts upon an abortion service in terms of unscheduled re-attendance rates and contraception provision at discharge.	590 (52 %) chose EMD. There was no significant difference in unscheduled re-attendance rates between EMD (*n* = 23, 4 %) and day case groups (*n* = 20, 4 %). There was no significant difference in the proportion of women in each group who left hospital with an effective method of contraception (*n* = 362, 61 % and *n* = 355, 60 % for EMD and day case groups, respectively).
(Blum et al. 2012) [55]	Medical abortion: Stanford University Hospital's OB/GYN clinic, Palo Alto, CA; Planned Parenthood Mar Monte, Sacramento, 2 clinics of the Family Planning Associates Medical Group, Chicago, USA	Quantitative descriptive: prospective clinical study and survey	429 women used a pregnancy test to determine baseline hCG on the day of mifepristone administration and follow-up hCG 1 week later. 189 women completed a survey.	To assess the effectiveness of a semi-quantitative home pregnancy test as a possible replacement for in-person follow-up after medical abortion.	The test identified the one ongoing pregnancy in the clinical study cohort. Sensitivity and specificity were calculated at 100.0 % and 97.0 %. The majority of participants in both the clinical study and the user comprehension survey found the test to be “very easy” or “easy” to use.
(Cameron et al. 2010) [41]	Medical abortion: hospital Edinburgh, Scotland, UK	Quantitative descriptive: prospective clinical study and survey	145 women chose to go home to abort, 69 % women completed questionnaires	To assess effectiveness and satisfaction of women with early medical discharge after abortion	The commonest reasons given for choosing to go home were: to get home sooner (53 %) and to be in the privacy of one’s own home (47 %), 81 % of the women stated that bleeding was either “as expected” (55 %) or “not as bad as expected” (26 %), and 58 % of the women stated that the pain was “as expected” (40 %) or “not as bad as expected” (18 %), 84 % of the women said that they would recommend this method to a friend.
(Cameron et al. 2012) [42]	Medical abortion: Royal Infirmary of Edinburgh, Scotland, UK	Quantitative descriptive: prospective clinical study and survey	476 opted for telephone follow-up	To evaluate telephone follow-up in terms of describing the numbers of women choosing to be followed up in this way, follow-up rates, efficacy of LSUP test for detecting ongoing pregnancies and women’s satisfaction	476 out of 619 women (77 %). opted for telephone follow-up, 4 women (1 %) attended the clinic before telephone follow-up because of pain or bleeding. A total of 410 (87 %) of the remaining 472 women were successfully contacted by telephone. Sixty women (15 %) screened ‘positive’, three of whom had ongoing pregnancies, and one woman falsely screened ‘negative’. The sensitivity of the telephone follow-up was 75 % [95 % confidence interval (CI) 30.1–95.4], and specificity was 86 % (95 % CI 82.2–89). The negative predictive value was 99.7 % (95 % CI 98.4–99.9), and positive predictive value was 5 % (95 % CI 1.7–13.7). All women surveyed (*n* = 75) would recommend telephone follow-up to a friend.
(David et al. 2007) [62]	Perm, Berezniki and Veliky Novgorod 20 health care sites (five maternity hospitals, six women’s consultation centres linked to those hospitals, three family planning clinics and six children’s polyclinics Russia	Quantitative descriptive: impact, pre- and post-intervention surveys	489 abortion clients in 2000, 559 in 2002 and 527 in 2003 surveyed in three facilities	To evaluate a post abortion care program to increase uptake of family planning	The project interventions appear to have extended the coverage of contraceptive counselling to nearly all abortion clients. Providers training resulted in increased, and possibly more effective, discussions about contraception with abortion clients. More than 80 % of post-abortion clients expressed not only an intention to use medical contraception but had identified their method of choice. Findings suggest that certain women conceive again soon after an abortion procedure and are more likely to abort again rather than try to prevent the unwanted pregnancy. Abortion is widely available and easily accessible, reducing the need for women to practice consistent and effective contraception, but most women say that they would prefer to prevent unwanted pregnancies through the use of modern contraception however these are either too expensive or reportedly fail due to poor quality.
(Dodge, Haider & Hacker 2012) [56]	Medical and surgical abortion: USA	Quantitative descriptive: simulated patient calls to services in the 5 most and 6 least abortion restrictive states using a survey	142 telephone calls were made to 48 non-provider facilities; 46 facilities were contacted 3 times and 2 were contacted twice	To determine the quality and quantity of referrals for abortion services from reproductive health care facilities that do not provide abortion services.	45.8 % of call resulted in a direct referral, 19.0 % resulted in an indirect referral, 8.5 % resulted in an inappropriate referral and 26.8 % resulted in no referral. Facilities in least restrictive states were significantly more likely to provide unprompted direct referrals (*p* = 0.006) and significantly less likely to provide no referral (*p* < 0.001) than facilities in most restrictive states, though these differences disappeared after prompting the staff member to provide a referral.
(Doran & Hornibrook 2014) [22]	Surgical abortion: New South Wales, Australia	Qualitative descriptive	13 rural women who had an early surgical abortion 5 months to 15 years’ prior	To identify factors that women experience in relation to their ability to access an abortion service and follow-up care	The main barrier rural women experienced was travelling relatively long distances to access an abortion clinic because of lack of services in their local area. Women with limited financial resources needed to borrow money for the procedure and associated costs of travel and accommodation. Women’s Health Centres provide a range of support and referral information. Lack of integrated care was reported.
(Esen et al. 2006) [43]	Medical and surgical abortion: TOP clinic South Tyneside Foundation Trust, England UK	Quantitative descriptive: prospective survey and examination of case notes	340 women requesting termination of pregnancy seen at the clinic in 2003	To evaluate the termination of pregnancy service in South Tyneside including our compliance with Royal College of Obstetricians and Gynaecologists termination of pregnancy guidelines	The number of referrals represented one-fifth of all births in our unit during the study period. Teenagers were the largest single group of women requesting termination of pregnancy and the majority were nulliparous. There were 85 women who were seeking a repeat termination of pregnancy. The RCOG minimum referral standard was met in 80 % of cases. A good number of women were unsure of their menstrual dates and only 5 % had used emergency contraception. A total of 96 % were either not using contraception, using condoms or taking oral contraceptives irregularly. A total of 50 % of the women attended hospital without a Certificate A being completed by the referring practitioner. Surgical termination was preferred over medical termination in the cohort of women who could exercise a choice.
(Finnie, Foy & Mather 2006) [44]	Medical and surgical abortion: South Durham in the North East of England, UK	Quantitative descriptive: survey and reviewed the case notes of women	Women attending two fertility control clinics general practitioners who referred women to these clinics	To identify service-related delays and barriers faced by women seeking abortion care	Of 210 women surveyed, 132 (63 %) responded. Of 107 referred by GPs, 16 (15 %) had to make a second appointment with another GP willing to refer them and 34 (32 %) waited two or more days to receive a date for their hospital appointment. The national standard waiting time of 3 weeks from first appointment with the referring doctor to the procedure was achieved for 56/127 women (44 %; 95 % CI, 35–53). Women rated global satisfaction, provision of information and staff interaction more highly in secondary than primary care. Of 170 GPs surveyed, 140 (82 %) responded; 33 (24 %) considered themselves ‘broadly anti-abortion’.
(Graham, Jayadeva & Guthrie 2010) [45]	Medical and surgical abortion: Hull and East Riding hospitals in Yorkshire, England UK	Quantitative descriptive: A retrospective audit of facility data	8,476 medical or surgical induced abortions undertaken at 14 weeks’ gestation or under	To assess the effectiveness of an integrated care pathway for delivery of evidence-based practice in abortion care.	100 women were re-admitted into the gynaecological wards of hospitals. Readmission rate was 1.2 %. The ICP showed that 97 % of women had chlamydia screening prior to the abortion; all women had a contraceptive discussion and 43 % left using a long-acting reversible method of contraception (LARC). However, data outside the care pathway was not documented, and hence the standard of care given on readmission was difficult to locate and variable in quality.
(Grindlay, Lane & Grossman 2013) [57]	Medical abortion: Planned Parenthood clinics USA	Qualitative in-depth interviews thematically analysed	25 women receiving medical abortion services (20 telemedicine patients and 5 in-person patients) and 15 clinic staff.	To evaluate patients’ and providers’ experiences with telemedicine provision of medical abortion.	Patients and providers cited advantages of telemedicine, including decreased travel for patients and physicians and greater availability of locations and appointment times compared with in-person provision. Overall, patients were positive or indifferent about having the conversation with the doctor take place via telemedicine, with most reporting it felt private/secure and in some cases even more comfortable than an in-person visit. However, other women preferred being in the same room with the physician, highlighting the importance of informing women about their options so they can choose their preferred service modality.
(Grossman et al. 2011) [58]	Medical abortion: Planned Parenthood affiliate in Iowa USA	Quantitative descriptive: prospective clinical cohort study and self-administered survey	Of 578 enrolled participants, follow-up data were obtained for 223 telemedicine patients and 226 face-to-face patients.	To estimate the effectiveness and acceptability of telemedicine provision of early medical abortion compared with provision with a face-to-face physician visit	99 % of telemedicine patients had a successful abortion was for (95 % confidence interval [CI] 96–100 %) and 97 % for face-to-face patients (95 % CI 94–99 %). 91 % of all participants were very satisfied with their abortion, although in multivariable analysis, telemedicine patients had higher odds of saying they would recommend the service to a friend compared with face-to-face patients (odds ratio, 1.72; 95 % CI 1.26–2.34). 25 % of telemedicine patients said they would have preferred being in the same room with the doctor. Younger age,
(Grossman et al. 2013) [59]	Medical abortion: Planned Parenthood affiliate in Iowa USA	Quantitative descriptive: Review of Iowa vital statistic data and billing data from the clinic system	17 956 abortion encounters 2 years prior to and after the introduction of telemedicine in June 2008	To assess the effect of a telemedicine model providing medical abortion on service delivery in a clinic system	The abortion rate decreased in Iowa after telemedicine introduction, and the proportion of abortions in the clinics that were medical increased from 46 % to 54 %. After telemedicine was introduced, and with adjustment for other factors, clinic patients had increased odds of obtaining both medical abortion and abortion before 13 weeks’ gestation. Although distance travelled to the clinic decreased only slightly, women living farther than 50 miles from the nearest clinic offering surgical abortion were more likely to obtain an abortion after telemedicine introduction.
(Gupta & Kapwepwe 2007) [46]	Medical abortion: Waltham Forest in England, UK.	Quantitative descriptive: A retrospective audit of facility data	1,257 abortions were undertaken in Waltham Forest in 2004/5	To evaluate a newly established service with an NGO	A 92 % completed abortion outcome was achieved. 58/63 completed EMA; 5/63 failed EMA.
(Hamoda et al. 2005) [47]	Surgical abortion: Aberdeen Royal Infirmary, National Health Service setting. Scotland UK	Quantitative descriptive: prospective clinical study and survey	56 women	To assess the feasibility, acceptability and efficacy of MVA under local anaesthesia for termination of pregnancy up to 63 days’ gestation	The mean (SD) gestation was 50 (9.4) days. A total of 55/56 (98 %) women had a successful procedure and did not require any further surgical or medical treatment. Fifty-five (98 %) women were satisfied with the procedure, 48 (86 %) said they would recommend it to a friend and 45 (80 %) said they would have the same method again in the future. Anxiety levels, as reflected by the visual analogue scales, showed a significant fall in anxiety scores following the procedure (*p* < 0.01).
(Jones & Jerman 2013) [60]	Medical and surgical abortion: All US states	Quantitative descriptive:	national sample of 8,338 abortion patients	To assess how far abortion patients travelled to a provider in 2008 and which groups were more likely to travel farther	In 2008, women travelled a mean distance of 30 miles for abortion care services, with a median of 15 miles. Sixty-seven percent of patients travelled less than 25 miles, and six percent travelled more than 100 miles. Controlling for other factors, women who lived in a state with a 24-h waiting period, women obtaining second trimester abortions, those who crossed state lines, and, in particular, rural women were more likely to travel greater distances relative to their counterparts. Women of colour were less likely to travel long distances compared to non-Hispanic white women.
(Kimport, Cockrill & Weitz 2012) [61]	Medical and surgical abortion: three abortion clinics located in the Midwest and south USA	Qualitative: thematic analysis	41 women who received an abortion	To understand impacts of abortion clinic structures and processes	The processes and structures of the abortion clinic necessitated by the realities of antiabortion hostilities lead some women to react negatively to the clinic experience in ways consistent with the social myth of the abortion clinic. Staff interactions can mitigate or alleviate these experiences.
(Lipp 2009) [48]	Medical and surgical abortion: National Health Service Trusts in Wales, UK	Quantitative descriptive: survey	All National Health Service Trusts	To establish current provision in termination of pregnancy	In the nine Trusts performing abortions, medical abortions accounted for 57 % and surgical abortions for 43 %. Doctors in training were involved in six Trusts. All but one Trust complied with referral times. Five Trusts provided a dedicated clinic. Written information provided prior to abortion varied in accessibility and quality. Choice of abortion within gestation bands was limited in some Trusts with some only providing medical termination. Essential abortion aftercare was performed by Trusts, whereas follow-up and counselling were less comprehensive.
(Mason 2005) [49]	Medical and surgical abortion: two Primary Care Trusts to a National Health Service-funded abortion clinic in the North West of England	Quantitative descriptive: survey	all clinic attendees from within the study area during a 6-month period	To investigate the referral process	90 % of the women were referred directly from the first health professional they consulted to the abortion clinic. Five percent of the women were either referred to another health professional or not referred anywhere. Twelve percent of the women had to wait longer than the 3 weeks recommended by the Royal College of Obstetricians and Gynaecologists guideline. In a minority of cases this wait extended up to 7 weeks. However, most women were satisfied with the length of wait, the health professional they consulted with and, in particular, the care they received at the abortion clinic itself.
(McKay & Rutherford 2013) [50]	Medical abortion: Peterborough City Hospital, UK,	Quantitative descriptive: survey at 24 h and 2 weeks following the procedure	127 women	To assess women’ s satisfaction with the home medical abortion service.	At 24 h, over 95 % of women who responded, agreed or strongly agreed that they felt prepared for the pain and bleeding that they experienced at home. At 2 weeks, 97.3 % of respondents felt that they had had enough information and knew what to expect, and were therefore satisfied with the procedure. Only 15 % of women were lost to clinical follow-up at 2 weeks. The majority of women are satisfied with the home medical abortion service. These high satisfaction rates are maintained at 2 weeks. Telephone follow-up 2 weeks after the abortion was safe and effective.
(Nickson, Smith & Shelley 2006) [23]	Medical and surgical abortion Victoria, Australia	Quantitative descriptive: multi-centre, cross-sectional observational study using a survey	All women accessing private services over a period of 12 weeks.	To investigate the extent and cost of travel undertaken by women accessing TOP services.	1,244 Australian resident respondents who resided in Victoria, 9.3 % travelled more than 100 km to access services. Teenagers were 2.5 times more likely than other respondents to travel further than 100 kilometres (km) (18.2 % compared with 7.8 %, *OR* = 2.5, 95 % CI 1.5–4.2, *p* < 0.001). Women originated from all Australian States and Territories except South Australia and 13.7 % were from Statistical Divisions other than Melbourne. More than one-third of respondents (41.3 %) chose their clinic because they were referred by a doctor or general practitioner.
(Norman, Hestrin & Dueck 2014) [67]	Medical and surgical abortion: British Columbia Women’s Hospital and Health Centre, Canada	Quantitative descriptive: A retrospective audit of calls	1998–2008 telephone calls	To review the toll-free pregnancy options service provision model for counselling and referral	Over 2000 women annually access service via the POS line, networks of care providers are established and linked to central support, and central program planners receive timely information on new service gaps and access barriers.
(Oliveras, Larsen & David 2005) [63]	Medical and surgical abortion: Hospitals in three cities in Perm, Berezniki, Veliky Novgorod Russia	Quantitative descriptive secondary analysis of data collected in a survey	489 abortion clients	To explore client satisfaction with abortion care	Client characteristics, in general, did not affect overall satisfaction though there were significant differences in overall satisfaction for unmarried versus married women (*OR* = 0 · 29, CI = 0 · 13, 0 · 63). Similarly, most characteristics of the abortion visit were not related to client satisfaction, although women who were awake for the procedure were less likely to be satisfied (*OR* = 0 · 37, CI = 0 · 16, 0 · 89). Information provided to abortion clients about self-care was the most important predictor of overall satisfaction for abortion clients (*OR* = 3 · 55, CI = 1 · 64, 7 · 69)
(Pillai et al. 2015) [51]	Surgical abortion: NHS Sexual Health Service, UK	Quantitative descriptive: A retrospective audit of facility data	1681 women	To assess the applicability, acceptability and cost implications of introducing MVA with local anaesthesia for fully conscious first-trimester termination of pregnancy	MVA was chosen by 305/1681 potentially eligible women. Forty percent had the procedure on the day they attended for assessment. 79 % gave a pain score of 3 or less out of 10. Complications occurred in six cases (2 %); these included cervical rigidity, a false passage, retained products of conception, bleeding (more than 200 ml) and one allergic reaction. Eighty percent of women chose to commence a long-acting reversible contraception (LARC) method at the time of MVA. Operating theatre utilisation was reduced by one termination list per week and cost savings of around £60 000 per annum were realised.
(Sharma & Guthrie 2006) [52]	Surgical abortion: Women and Children’s Hospital, Hull Royal Infirmary, Hull, UK	Quantitative descriptive: A prospective audit of facility data and staff survey	12 women <10 weeks’ gestation at the time of termination, February–March 2004, 23 staff	To evaluate nurse led telephone booking service and local anaesthetic outpatient surgical termination of pregnancy	Demand from referrers for the telephone booking clinic was greater than could be accommodated by the service. Telephone consultation was popular with patients as it was carried out at their convenience in their homes, and with staff as it reduced clinic assessment time. Some staff members felt that consulting over the telephone affected their assessment of the patient’s emotional status. Outpatient LA-STOP seemed well accepted by both staff and patients as it offered patients a convenient and safe method of early abortion. A preliminary costing indicated a net saving plus increased service capacity.
(Silva & McNeill 2008) [64]	Medical and surgical abortion: throughout New Zealand	Quantitative descriptive: analysis of Census data and Statistics NZ data, calculation of distances from site of referral	Nationwide TOP service information from 2006	To assess geographic accessibility to first trimester termination of pregnancy	Women who live in regions that do not offer local TOP services must travel on average 221 km to access TOP services. This equates to an average return-trip distance of 442 km. Three of the five regions that do not have local TOP services available have a higher than average proportion of Maori population
(Silva, McNeill & Ashton 2011) [65]	Medical and surgical abortion: Nine of a total of 13 first trimester clinics throughout New Zealand	Quantitative descriptive: A prospective audit of facility data and survey of women	2,950 patients attending nine abortion clinics between February and May 2000	To identify the factors affecting the timeliness of services in first trimester abortion service	Women who went to private clinic had a significantly shorter delay compared to public clinics. Controlling for clinic type, women who went to clinics that offered medical abortions or clinics that offered single day services experienced less delay. Also, women who had more than one visit with their referring doctor experienced a greater delay than those who had a single visit. The earlier in pregnancy women sought services the longer the delay. Women’s decision-making did not have a significant effect on delay.
(Snook & Silva 2013) [66]	Medical and surgical abortion: community-based services in a high-deprivation district health board New Zealand	Quantitative descriptive: A prospective audit of facility data	180 women who had an abortion in 2010	To describe the services developed and assess safety and timeliness for the first year of service.	Eighty-two percent of locally provided abortions in 2010 were medical abortions, completed on average less than 2 days after referral to the service. One percent of patients experienced haemorrhaging post abortion, and 4 % had retained products. These rates are within accepted standards for an abortion service.
(Tupper & Andrews 2007) [53]	Medical abortion: Morecambe Bay Primary Care Trust UK	Quantitative descriptive: A prospective audit of facility data	171 women referred	To report on setting up and running a new outpatient service for early medical termination under 7 weeks’ gestation.	Of 171 patients referred in the first year, 148 were offered an appointment and 100 women completed outpatient treatment for medical termination under 7 weeks’ gestation

Forty-one papers were appraised using the Critical Appraisal Skills Programme tool for qualitative research [39] and Pluye et al.’s [40] scoring system was used to assess the non-experimental and mixed method studies. Fourteen items were discarded due to methodological concerns. The lead author first appraised the papers using the checklists of both tools which were independently verified by the DB and ES.

Data were extracted from the twenty-nine papers and first described according to characteristics of the research (e.g., primary author, year of publication, study setting); research participants (age, gender, and socio-demographic data); study aim; study design, and findings (see Table 3). The conceptual framework outlined at Table 1 was then applied to identify the dimensions of access and demand or supply perspective. A content analysis of the extracted text relating to each dimensions of access was performed by the first author and then discussed with all authors. The questions aligned with each dimension of access as were used to interrogate the findings of the paper. We then identified evidence of processes and outcomes that contributed to the ability of the service to increase access to abortion, as well as the constraints, to provide insight into how access to services could be improved in Australia.

## Results

Twenty-nine papers were included in this review (see Table 3). Fourteen papers were from the United Kingdom (UK) [41–54], seven from the United States (US) [55–61], two from Russia [62, 63], two from Australia [22, 23], three from New Zealand (NZ) [64–66] and one Canadian paper [67]. Of these papers all were quantitative except for three qualitative studies [22, 57, 61].

We categorized the papers according to the aspect of the abortion service under study, outcome measures, dimensions of access covered (see Table 4) and their focus on supply, demand or both (see Table 5). Of these, six papers focus specifically on service delivery related to aspects of surgical termination of pregnancy (STOP) while nine papers were concerned with medical termination (MTOP). Other papers describe both types of abortion with service related outcomes according to the components of care [48, 63], approach to service integration [45], referral, booking and counselling [43, 44, 49, 56, 67] and the type of facility where the services are provided [23, 46, 60, 61, 64–66]. Of the access dimensions covered, least covered were adequacy and affordability with supply focused papers gaining more attention than those solely concerned with demand side issues. The results of the analysis of the findings section of the 29 papers as per the questions generated from the dimensions in Table 1 are described below. We have specifically reported factors that have been identified to enhance access service delivery and those that have been found to constrain access to services.

**Table 4:** Access Dimensions Covered across papers in the review

	*Availability*	*Accessibility*	*Affordability*	*Adequacy*	*Acceptability*	*Quality*
(Astle, Cameron & Johnstone 2012) [54]						√
(Blum et al. 2012) [55]					√	
(Cameron et al. 2010) [41]					√	√
(Cameron et al. 2012) [42]					√	
(David et al. 2007) [62]	√					√
(Dodge, Haider & Hacker 2012) [56]		√				
(Doran & Hornibrook 2014) [22]	√	√	√		√	
(Esen et al. 2006) [43]		√				√
(Finnie, Foy & Mather 2006) [44]		√			√	√
(Graham, Jayadeva & Guthrie 2010) [45]						√
(Grindlay, Lane & Grossman 2013) [57]	√	√	√	√	√	
(Grossman et al. 2011) [58]					√	√
(Grossman et al. 2013) [59]	√	√				
(Gupta & Kapwepwe 2007) [46]	√					√
(Hamoda et al. 2005) [47]					√	√
(Jones & Jerman 2013) [60]		√				
(Kimport, Cockrill & Weitz 2012) [61]		√		√	√	
(Lipp 2009) [48]	√					√
(Mason 2005) [49]		√				
(McKay & Rutherford 2013) [50]					√	
(Nickson, Smith & Shelley 2006) [23]		√	√		√	
(Norman, Hestrin & Dueck 2014) [67]	√				√	√
(Oliveras, Larsen & David 2005) [63]				√	√	
(Pillai et al. 2015) [51]			√		√	
(Sharma & Guthrie 2006) [52]	√		√		√	
(Silva & McNeill 2008) [64]		√				
(Silva, McNeill & Ashton 2011) [65]		√				
(Snook & Silva 2013) [66]	√	√				√
(Tupper & Andrews 2007) [53]	√		√			√

**Table 5:** Approach to abortion service, outcomes, access dimension and perspective

	Aspect of abortion service under study	Outcome measures	Supply and/or demand perspective
	Outpatient/home based/early medical discharge (MTOP)		
(Astle, Cameron & Johnstone 2012) [54]		Unscheduled re-attendance rates, contraception provision at discharge rate	Supply
(Cameron et al. 2010) [41]		Women’s satisfaction	Demand
(McKay & Rutherford 2013) [50]		Women’s satisfaction	Demand
(Tupper & Andrews 2007) [53]		Referral rates, completion rates	Supply
(Blum et al. 2012) [55]	Home-based follow up using semi quantitative pregnancy test	Sensitivity and specificity of a pregnancy test and women’s usability	Supply–demand
(Cameron et al. 2012) [42]	Home-based follow up using nurse-led telephone calls	follow-up rates, efficacy for detecting ongoing pregnancies and women’s satisfaction	Supply–demand
	In patient/clinic based telemedicine (MTOP)		
(Grindlay, Lane & Grossman 2013) [57]		Women and staff experience and satisfaction	Demand
(Grossman et al. 2011) [58]		Completion rates, women’s satisfaction, preferences and recommendations	Demand
(Grossman et al. 2013) [59]		Abortion rates, distance women travelled	Supply–demand
	Outpatient STOP		
(Doran & Hornibrook 2014) [22]	Outpatient STOP	Experience of access to facility	Demand
(Hamoda et al. 2005) [47]	MVA under local anaesthesia	Completion rates, satisfaction, anxiety levels	Supply–demand
(Pillai et al. 2015) [51]	MVA under local anaesthesia	Completion rates, Pain, complications, contraception uptake, cost	Supply–demand
(Sharma & Guthrie 2006) [52]	MVA under local anaesthesia and telephone booking service	Number abortion completions, of telephone consultations, referrals, and staff satisfaction	Supply
	Abortion care components		
(Lipp 2009) [48]	STOP and MTOP services	Service provision types	Supply
(Oliveras, Larsen & David 2005) [63]	MVA STOP, MSTOP, dilation and curettage, post-abortion family planning counselling	Women’s satisfaction	Demand
(David et al. 2007) [62]	Post abortion care program	Contraception counselling delivered	Supply
	Care delivery program type		
(Graham, Jayadeva & Guthrie 2010) [45]	Integrated care pathway, MTOP and STOP	Re-admission rate, contraception advice received and uptake	Supply
	Referral, booking and counselling		
(Dodge, Haider & Hacker 2012) [56]	Referral advice	Referral rates	Supply
(Esen et al. 2006) [43]	Referral process	Waiting times	Supply
(Finnie, Foy & Mather 2006) [44]	Referral process	Waiting times within the pathway to induced abortion, women’s rating of care, GPs’ attitudes and self-reported practice	Supply–demand
(Mason 2005) [49]	Public service referral process	Length of wait, number of professionals involved, women’s satisfaction	Supply–demand
(Norman, Hestrin & Dueck 2014) [67]	Toll free options service provision	Use rate	Supply
	Facility type		
(Gupta & Kapwepwe 2007) [46]	NGO	Completion rates	Supply
(Jones & Jerman 2013) [60]	Public and private clinics	Distance travelled	Supply
(Kimport, Cockrill & Weitz 2012) [61]	Public and private clinics	Experience of facility structure and process	Demand
(Nickson, Smith & Shelley 2006) [23]	Private services	Distance travelled, money and time expended undertaking travel, and reasons women chose particular clinics	Supply–demand
(Silva & McNeill 2008) [64]	Public and private clinics	Number of services, return trip driving distance	Supply
(Silva, McNeill & Ashton 2011) [65]	Public and private clinics offering MTOP vs single day services	Timeliness	Supply
(Snook & Silva 2013) [66]	Community based services	Safety and timeliness	Supply

### Availability

The availability of both MTOP and STOP services at facilities was found in one study to increase access. Tupper et al. describe the development of a new MTOP service in the UK to address a gap in the public sector provision of abortion; and to increase the timeliness and choice of type of first trimester abortion for women in an area where only STOP had previously been available and MTOP via an out of area private provider [53]. This retrospective audit of the first 12 months of the service indicate that it went some way towards addressing the need for an increase in the availably of MTOP and meeting the Department of Health targets on TOP to reduce waiting times and increase the number of women provided with an abortion under 10 weeks’ gestation. The findings show that all available appointments were filled in the first week that the service was opened however some patients had to be referred in the first 8 months due to a lack of staffing capacity. The service was therefore extended to accommodate the larger than anticipated demand resulting in lower numbers of onward referrals. Another UK study evaluated a newly established, public and NGO sector MTOP service to increase women’s choice in an area where STOP was only available. However the impact of the new service was not reported other than the numbers of successful early MTOP procedures [46].

Three papers in the review outline the NGO, Planned Parenthood’s efforts to increase the availability of MTOP in the one State in the US by providing medical abortion via telemedicine at clinics without an on-site physician [57–59]. Women noted the service enabled them to undergo the procedure sooner without waiting for an appointment which ensured the availability of MTOP as per the gestational age limit. Staff noted that the service was able to achieve a greater reach and offer more choice for abortion, as well as access to timely same day treatment. Staff stated that they found the process of introducing the service useful in building their skills and ability to provide new services [57]. There was an increase in the proportion of medical abortions in the clinics from 46 to 54 %. Clinic patients had increased odds of obtaining both MTOP before 13 weeks’ gestation after the introduction of telemedicine [58, 59].

Another paper examined the use of a telephone booking service to increase the availability abortion via manual vacuum aspiration (MVA) under local anaesthesia in a local English hospital [52]. The study established that demand from referrers for the telephone booking clinic was greater than could be accommodated by the service [52]. However, substituting two doctor assessment clinics of 10 patients for two clinics of six patients, supported by 2.5 nurse telephone clinics, was able to increase the service capacity by 25 %.

Other studies in the review identified the impact of the health workforce on the availability of abortion services. A shortage of doctors, particularly female doctors who were willing to perform abortions was noted by staff of women’s health centres in rural Australia. In addition, staff cited a shortage of rural general practitioners as impacting upon the availability of referral points and follow up care [22]. A study of GPs (No = 126) in the north east of England UK found that approximately one-third surveyed did not provide on-site pregnancy testing (52; 37 %) and believed they had insufficient information about abortion services (43; 32 %) [44]. It is suggested that these factors affected service availability.

One study from NZ showed that a newly established community-based abortion clinic in one district health board (DHB) developed to provide local service provision and prevent out-of-region referrals was able to contribute to addressing the need for increased availability. Of the 180 women from the DHB who had an abortion in 2010, 81 % (*n* = 145) had an abortion locally, while the remaining 19 % went outside the region for the service [66]. However, service availability did not appear to be equitable as three of the five regions that did not have abortion services were found to have a higher than average proportion of a largely disadvantaged Indigenous Maori population.

### Accessibility

Two studies in the review investigated the use of technology to address accessibility issues, in particular to eliminate travelling time and overcome geographical barriers to abortion services. Qualitative interviews with women who selected a MTOP telemedicine clinic stated that they did so due to logistical concerns as they were unable to access a clinic close by even if they preferred to do so [57]. Women also stated that this saved them time, the stress of driving to a facility and the need to take time off work. The researchers found that women living more than 80 km from the closest facility that offered STOP were more likely to obtain an abortion after the introduction of telemedicine [58, 59].

Accessibility in the studies reviewed is manly discussed in relation to constraints. The time and travel distance required to access abortion services is examined in a number of studies included in the review with accessibility for rural women and adolescents more disadvantaged according to three papers. The statistical model in Jones et al. US study showed that having an early abortion was not associated with increased travel but that women who lived in rural areas and those who lived in a state with a 24-h waiting period were more likely to experience increased travel [60]. In Australia, Nickson found that there were limited rural services and adolescents were 2.5 times more likely than other women to travel more than 100 km (18.2 % compared with 7.8 %, OR = 2.5, 95 % CI 1.5–4.2, *p* < 0.001) [23]. Interstate travel in order to access an abortion was noted in two Australian studies [22, 23] with public transport largely unavailable [22].

A qualitative study of women’s experiences of three abortion clinics in the US noted that women sometimes made a decision to travel a greater distance to specific clinics to avoid protesters and the associated stress [61]. Avoiding protesters was also noted by women as one reason for selecting a particular clinic in an Australian however, most women selected the clinic on where they obtained their abortion on proximity to their place of residence [23].

Several studies were included in the review revealed issues regarding referral and the accessibility of services. Dodge et al. [56] studied referral to abortion services in America from staff at facilities that did not provide abortion showing that less than half of the women received direct referrals. Women who did not prompt the staff for a referral during a call to facilities in States with less restrictive abortion laws were significantly more likely to be provided with direct referrals (*p* = 0.006) than women who called facilities in more restrictive states. Accessibility was also hampered according to women in an Australian study by slow referrals from health providers [22]. In the UK it was found that referral for abortion was mostly from general practitioner doctors (GP) [44]. However, a review of clinic records in one area in the UK shows that about half of the patients were referred without a Certificate “A” being completed by the referring GP, the reasons for this in some cases was due to issues doctors had with abortion and their conscientious objection to TOP. It was suggested that this may impact upon the speed at which women are able to obtain and abortion [43]. Women in NZ who had more than one visit from their referring doctor also experienced a longer delay to receive an abortion than those who had a single visit [65].

Timeliness of referral was also examined in studies in relation to established recommendations from the UK Royal College of Obstetricians and Gynaecologists (RCOG). Finnie et al. found that referral targets were largely achieved with 52% of women seen within 48 h of making an appointment [44]. In addition, the majority (97 %) of women had abortion procedures within 14 days of clinic appointments [44]. Mason’s study found that twelve percent of the women had to wait longer than the 3 weeks, exceeding the RCOG’s recommendations regarding referral [49]. The survey of GPs found they referred girls under 16 years who requested an abortion with a parent or guardian [44]. Fewer (72; 52 %) reported that would refer unaccompanied girls under 16.

Services factors were found to affect the speed at which women were able to gain an abortion in NZ however the rationale for this is not clear. Silva et al. found that women who obtained their abortion from a private clinic had a significantly shorter delay compared to those who attended public clinics. A shorter delay was also experienced by women who received care from facilities that provided MTOP or single day services and for those women with a lower gestational age [65]. Another NZ study also found that women choosing MTOP had a shorter wait than those obtaining a STOP [66].

### Affordability

Some of the studies included in this review examined costs from the perspective of the health system, health professionals and the individual women. In one English primary health care trust Tupper et al. found that a new MTOP service contributed towards cost savings in the UK National Health Service (NHS) budget [53]. The authors compare the cost of their services with those of the ‘Payment by Results’ national tariff of fixed prices that reflect national average prices for hospital procedures. Costs per case in the new service are outlined as £157 GBP at set up which was estimated to be £85 GBP within 5 years of operation. This compares favourably to the £498 ‘Payment by Results’ per case on for surgical termination and £423 for medical termination.

A study undertaken in a different English primary health care trust a year earlier noted that the cost of a MTOP or a STOP (day case) ranged from £462 to £578 per patient [52]. Sharma et al. study of a pilot local anaesthetic outpatient STOP service found that the cost could be reduced to £366 per patient. However, if this involved outpatient consultation the cost was £217 per patient which could be further reduced to £177 if the nurse telephone clinic was used. The nurse telephone clinic reduced the time needed by the doctor to assess each patient, which increased the number of patients that could be seen per clinic. Recent research in the UK also examining a local anaesthetic outpatient STOP service found that a cost savings was made of approximately £60000 per year and that the operating theatre use was reduced by one termination list per week [51].

Interviews with American women accessing a MTOP telemedicine clinic [57] explained that they selected the as it saved them money that they would have had to spend on travel. For some women obtaining a STOP in Australia was very expensive, particularly rural women who in one study said they had borrowed money to cover not only the abortion fee but pay for travel, accommodation and additional childcare costs [22].

### Adequacy

The adequacy of abortion services in terms of the organization of clinics and their hours, of operation, cleanliness and administration processes was the subject of interest in three studies in the review. Interviews with US staff and women revealed that the telemedicine MTOP service offered services more frequently and with a wider range of times available to women than clinic based services. Prior to the telemedicine service staff noted that women could only see doctors on a particular day of the week or month that they visited the clinic, whereas telemedicine enabled women to schedule their consultation any day of the week if needed [57]. Abortion clients from a USAID funded Women and Infant Health Project who were surveyed in three cities in Russia rated level of comfort and hygiene. Ratings of comfort had the greatest variation with 41 % of clients rating comfort as good, 47 % as fair and 11 % as poor. Less than two per cent of women rated their satisfaction with hygiene as poor while 69 % reported satisfaction with hygiene as good, [63]. Interviews with women obtaining abortions from three clinics in the US described women’s experiences of antiabortion protesters outside and the clinics’ security measures in response [61]. The findings show that both experiences served to increase women’s feelings of stigmatisation. Women described being “buzzed in” passing through metal detectors and paying in cash that made the process seem impersonal and illicit.

### Acceptability

Four of the six papers in this review, that describe the acceptability of services that enable women to terminate their pregnancy at home rather than return to the hospital or clinic after a MTOP, report on women’s acceptability of this practice. A survey of 100 women in the UK found that women chose to go home as soon as possible after the procedure (53 %) and to be in the privacy of their own home (47 %). The majority of women did not regard the amount of pain or bleeding to be a considerable issue [41]. These findings concur with another survey of 127 women who reported that they were adequately prepared for the amount of bleeding and pain they experienced at home [50]. Women in both surveys were satisfied with the procedure with most women in Cameron et al. research (84 %) stating they would recommend home medical abortion [41].

Blum et al. research in the US examined women’s acceptability of a test to detect continuing pregnancy at home after their MTOP. The majority of women surveyed found the test easy to use with two thirds (58.1 %, *n* = 190) correctly identifying the need to return to the clinic based on the home test reading measures of serum human chorionic gonadotropin being the same or higher than their baseline level [55].

When offered, Cameroon et al. [42] found telephone follow up and self-performed pregnancy testing after their MTOP was more popular with women in the UK up to 9 weeks gestation than return visits to the hospital (476 out of 619 women or 77 %). Women stated that this was more convenient, less stressful and reduced the need for travel. The majority found the follow up calls from nurses re-assuring and would likely recommend this approach to others. Most women also expressed their preference for a low-sensitivity urine pregnancy test at 2 weeks after their MTOP (97 %). However, if given a choice the majority indicated a preference for self-assessment without a follow-up phone call (52 %) while 43 % stated that they would have been ‘unlikely’ to choose self-assessment.

In contrast to women in the study by Grossman et. al. [58] women in Grindlay et al. research paper [57] reported that they were not comfortable with the telemedicine approach to MTOP. These women felt it did not enable them to access other reproductive health services at the same time and that they were unsure about the service as it had not been recommended and was unknown by family and friends. Despite this many women who experienced the telemedicine consultation stated that they were satisfied and comfortable with a video communication as it was already part of their daily lives. Women noted that remote service provision reduced stigma and embarrassment and felt less intimidated. However, others felt that they would have preferred having a personal interaction with a provider and were concerned about privacy issues with a webcam. Most staff found telemedicine acceptable to deliver to women noting issues with detecting women’s Rhesus (RH) status. which led to delays in accessing the service. National guidelines recommend the administration of anti-D immunoglobulin to Rh (D) negative women within 72 h of an MTOP in order to prevent maternal sensitisation and an adverse effect on a future pregnancy [68, 69]. In the Grindlay et al. study, new providers of telemedicine based abortion services lacked the capability for on-site testing resulting in additional communication with women and third parties in order to establish their Rh status prior to the clinic visit.

Three studies included in this review included an examination of women’s acceptability of early STOP services in particular MVA under local anaesthesia. One study found women experienced little pain with 79 % giving a pain score of 3 or less out of 10 [51]. Women were found to recover quickly from their anxiety after their abortion with visual analogue scales, indicating a significant fall in anxiety scores following the procedure (*p* < 0.01). [47]. Women were also satisfied with the procedure in both studies. Eighty-six % of surveyed women said they would recommend it and 80 % said they would have the same method again in the future [47]. Sharma et al. survey [52] found that staff perceived the patients to be satisfied, “reassured” and “relaxed” and that the provision of a local anaesthesia gave women a choice compared with a general anaesthesia. Telephone consultation was popular with patients according to staff as it was carried out at their convenience in their homes, and with staff as it reduced clinic assessment time. Staff also noted that the telephone booking service with women was acceptable to patients who spent less time in the clinic but that it was more difficult to assess a women’s emotional state.

Abortion clients from a USAID funded project surveyed in three cities in Russia were largely satisfied with services received from health professionals Information provided to abortion clients about self-care was the most important predictor of overall satisfaction (OR = 3.55, CI = 1.64, 7.69). How much the client paid for the abortion did not affect satisfaction with respect to any aspect of care [63]. Despite some women having to wait longer than the 3 weeks recommended by the RCOG guidelines, or in a few cases up to 7 weeks, most women said they were satisfied with the service they received [49]. Women in a survey in the Australian State of Victoria cited the reasons for selecting a private clinic to have their abortion including the reputation of the clinic, quality of information received, as well as good staff and service [23].

Rural Australian women described dissatisfaction with the attitudes of GPs who delayed referral by not providing information concerning options for self-referral. These women stated the need to reduce stigma and negative attitudes to improve access to abortion [22]. American women described the impersonal attitudes of staff and that the denial of support persons for security reasons added to the stigmatisation of the experience. However women’s satisfaction was heightened by non-judgemental staff and the presence of a patient advocate [61]. Finnie et al. surveyed women in the UK accessing MTOP and STOP in the UK who overall rated satisfaction, provision of information and staff interaction more highly in secondary than primary care. Women noted that their GPs anti-abortion views were made known to them and although they were referred were provided with very little information concerning their options [44].

### Quality

Quality standards for abortion care were noted in several studies in relation to professional standards, national standards set by the government and local area health standards, as well as women’s views of the standard of quality care.

The standards of the RCOG in the UK were referred to in comparison with the results in five studies. In the paper by Essen and colleagues the RCOG minimum referral standard of women seen by a doctor within 2 weeks of referral was found to have been met in 80 % of cases. Of the 340 women in the study 111 patients had their TOP performed within 7 days of their consultation with a doctor in the hospital which was noted as the RCOG ideal standard [43]. The national RCOG standard waiting time of 3 weeks from first appointment with the referring doctor to the procedure was achieved in Finnie et al. study for 56 of 127 women (44 %; 95 % Cl, 35–53) [44]. In an earlier study the numbers were less with twelve percent of the women having to wait longer than the 3 weeks recommended by the RCOG guideline. In a minority of cases this wait extended up to 7 weeks [49]. Lipp’s investigation into services in nine Primary Health Trusts in Wales found that one Trust did not comply with referral times of 3 weeks as per clinical guidelines due to service constraints and five of 10 Trusts provided a dedicated assessment clinic as advised for women requesting TOP [48]. Lipp et al. also assessed the quality of written information provided to women prior to abortion in relation to RCOG guidelines that dictate that verbal information should be supported by written impartial, printed material [70]. Five Trusts included written information that was given to women during the initial consultation and on discharge. The information varied in both content and presentation and all material outlined the risks of complications. Tupper et al. [53] note in their evaluation of a new service that the RCOG standards and competences in providing abortion services were observed but no detail of this is provided. One NZ study compared their results of MTOP complications with the standards set by RANZCOG. Complication rates were very reported to be low and within the RANZCOG standards [66].

National standards were cited in a study from Scotland in the UK. The authors audit of facility data noted that National Health Service (NHS) Quality Improvement Scotland standards for sexual health services, which recommend that at least 60 % of women leave an abortion service with an effective method of contraception, was achieved [54].

Graham et al. [45] investigated the use of an integrated care pathway (ICP) in abortion care as a way of incorporating local and national guidelines into everyday practice and managing clinical risk while meeting the requirements of clinical governance in two hospitals in the UK. The researchers undertook an audit of facility data and found that in this context clinical records are a useful tool for high quality record-keeping and to ensure that all women receive the same standard of pre-assessment care. The integrated care pathway (ICP) approach to the provision of quality of MTOP and STOP was developed for two hospitals in the UK and involved a checklist that included STI screening, treatment and partner notification, prophylactic antibiotics at time of abortion, provision of contraception counselling and supply. The checklist also includes readmission investigations such as temperature taking, full blood counts and ultra sound. The ICP evaluation showed that 97 % of women had chlamydia screening before the abortion; all women had a contraceptive discussion and 43 % left using a long-acting reversible method of contraception [45].

## Discussion

This review has identified the dimensions of access to surgical and medical abortion from, clinic or hospital-outpatient based abortion services, as well as new service delivery approaches utilising a remote telemedicine approach. A range of factors, mostly from studies in the UK and US were found to facilitate improved access to abortion. In particular, flexible service delivery approaches that provide women with cost effective options and technology based services. These could provide possibilities for the Australian health system. However, it is not clear how many services in Australia provide a choice of MTOP or STOP with the situation largely dependent upon the will of private services.

Services that provided women with the choice of either MTOP or STOP [53] and the establishment of nurse-led telephone consultation and outpatient services for STOP [51, 52] in this review were found to be cost effective, in demand and the uptake high. Home based MTOP with pregnancy testing and telephone follow up was also reported to be acceptable to women [41, 42, 50, 55]. MTOP delivered by telemedicine was reported to have improved the availability and timeliness of abortion most women found it acceptable, reduced costs to the women and reduced the stress associated with the procedure [57–59]. A private company called the Tabbot Foundation in Australia is trialling the use of video and skype consultations for MTOP that reduces the need for a health services visit except for required tests, or in the case of complications. All of the 303 women involved in the trial at the end of 2015, gave the service a very high rating [71]. MTOP based telemedicine may, as in the American studies in this review, increase the availability of MTOP in Australia, including for women living in rural and remote settings. This could reduce costs associated with long distance travel and enable a timelier delivery of MTOP. In Australia, unlike in the American context, a face to face clinic visit for telemedicine MTOP is not a requirement but and women still need to obtain a blood test to check their rhesus status and an anti-D immunoglobulin injection if Rh negative [72].

The review identified a number of standards, recommendations and targets that provided services and providers with guidance regarding the quality of abortion care including the UK Department of Health targets [53] and RCOG recommendations regarding referral and waiting times [43, 44, 49]. The papers in this review also noted provider competences outlined by RCOG [53] which include the requirement that women leave an abortion service with appropriate information and an effective method of contraception [54]. These standards may provide RANZCOG and the Royal College of General Practitioners with insights to improve practitioner competence in addition to the standards associated with the rate of complications noted in one paper. Targets to reduce waiting times could also be applied nationally in Australia, as well as at State and Territory level, however this would be difficult due to the variability of current service provision. Targets could be applied to public provision but such services may not be apparent and available at all public facilities. Assessing progress on targets would require comprehensive and coordinated data collection that does not currently exist across Australia. The collection of abortion data should be alongside other reproductive health indicators and service evaluation. One such approach that might offer guidance are the input, process, outcome and impact indicators recommended by experts at an International workshop on reproductive health indicators and database development [73].

Difficulties accessing abortion services were noted for particular populations in this review, in particular for adolescents and women with limited financial means [22, 23, 60, 66]. Little is known about access to abortion services for adolescents who face issues of confidentiality and often negative attitudes of providers [74]. In Western Australia adolescents have been found to have the highest abortion rates [75] which is likely to reflect rates in other States and territories, in line with global figures [76]. Research is therefore needed to ensure that the needs of the highest users of such services are best addressed.

Although not mentioned by the research in this review, other studies have noted higher rates of abortions among migrant women in HIC [77] who face barriers such as poor access to information about contraception, difficulty paying for services, a history of trauma and abuse, and fear of deportation [78]. Sexual and reproductive ill health disproportionately affects migrant and refugee women in Australia and hence is an area of high need requiring attention [79]. In Australia, significant barriers have been documented regarding the access of refugees and migrants to primary health care [80] and recommendations for improving access to general practice have been put forward however, these do not include reproductive health issues such as abortion [81]. Migrants and refugees may also have different expectations of abortion based upon their experiences in their countries of origin. Access for refugees in detention is even more challenging as demonstrated by a recent case of a Somali asylum seeker detained by the Australian Government on the Island of Naru [75].

In our review the descriptions of services were largely public in the UK, NZ, Canada and Russia and private in US and Australia which reflects current service-delivery models in these countries. The model of a public private partnership only appeared in one UK-based study [46]. This difference dictated the papers focus on affordability. Where the cost was born by the state the discussion focused on health systems savings and efficiency, while costs on an individual level featured in the American and Australian papers where women largely access services through private clinics. We found little information concerning ways to best support women from lower socio economic backgrounds with payment if services were unavailable in the public system. While in Australia the universal health system Medicare reimburses costs for STOP and MTOP services, the lack of access through the public system in most states and territories means that women need to access private services leading to an out of pocket gap payment, which can be prohibitive for some women.

Apart from location of services, often concentrated in urban areas, availability is also determined by availability of trained personnel. Staff shortages were noted in papers in our review [22, 53] in the UK and Australia, as well as low knowledge about abortion [44]. Gynaecology and general practice training pathways are variable in some countries [82] and may not include abortion provision. Therefore gynaecologists and GPs must seek additional training to become STOP or MTOP providers, either in their own practices, or within private clinics. In Australia, despite the availability of training for GPs to provide MTOP in their own practices, the uptake of this training and translation into service delivery appears to be relatively low [83]. In the US leadership, the commitment of medical faculties, and the support of professional bodies have been cited as essential components of routine abortion training to increase service provision [84]. This highlights the leadership role of Australian medical, nursing, midwifery and pharmacy faculties and associated professional associations in undertaking to increase provision in public facilities. RANZCOG has developed a training program for its fellows and associates and produced a comprehensive resource for health professionals covering both STOP and MTOP procedures [85]. Task shifting with provision of STOP and MTOP services by, for example registered nurses, is also acceptable to women as noted in this review [52] and is not associated with a higher risk of complications. In Australia task shifting is in its naissance with nurses involved in supporting abortion care for women, however there have been no evaluations of such services. This is in contrast to middle and low income countries where nurses and other mid-level cadres have been found to perform MVA as safely and effectively as doctors in India and Nepal [86, 87].

Studies in the review noted that the anti-abortion views of doctors had slowed the referral process and that GPs made their views known to women [22, 43, 65]. Addressing conscientious objection and, more broadly, provision of sensitivity training for personnel linked to service delivery or referral pathways is critical. Practical training as well as sensitivity training is vital for all levels of service provider including gynaecologists, GPs and other primary care providers, pharmacists and ancillary staff. Our review did not provide insight into how such training should be undertaken and its impact upon access to services. However, doctors have been found to value teaching about the social issues surrounding abortion, as well as clinical ones [82]. Suggestions have been made by Kaposy in Canada to allow doctors conscientious objection while also ensuring access. This includes clear advertisement of objection at services and regulation by professional associations to prevent women experiencing referral delays, the refusal of information, or deliberate misinformation [88].

Legal restrictions in different Australian and US states were noted to affect referral and travel in two studies in the review [22, 56]. Variations in the legal status of abortion can also create confusion for providers and women and may present unnecessary barriers to service provision. While there are no barriers to the provision of abortion in public hospitals in Australia when performed within the state or territory-based legal framework, with the exception of South Australia, they appear to be rarely performed in this setting [89]. The lack of abortion services may relate to workforce issues and a lack of leadership, as well as the values of faith based hospital services. While most public hospitals will provide abortions, in certain circumstances hospital policy may impose gestational limits and there is generally no transparent central booking system for women or their GPs to access their abortion services. Kaposy highlights approaches Canada has taken to ensure public access that may be transferrable to Australia states and territories. These measures include national decriminalization and the designation of 33 public hospitals as abortion providers by the government of British Columbia [88].

Only one study in the review specifically examined an integrated care pathway [45]. Comprehensive abortion care incorporates the management of complications, provision of STI testing and treatment, as well as provision of contraception. The integration of abortion services within family planning services and/or sexual health services or conversely integration of family planning/sexual health services within abortion services is important in relation to reducing repeat abortions [90]. Experiences of integrating comprehensive abortion care into health services in HICs are difficult to locate in the literature despite many services, such as those in Scotland, in existence [91, 92]. Studies from LMIC show that it is feasible [93, 94] which may provide lessons for wealthier countries.

## Conclusions

Clear ways forward for abortion service delivery in Australia include the provision of choice and flexible options for women, in conjunction with appropriate use of technology and the application of standards to ensure universal access and quality abortion services for all women. The paucity of evidence concerning access to abortion services in Australia, particularly the dimension of availability, adequacy and quality, shows that more research is needed in this area. Of note is a lack of evidence about demand for abortion services including the needs of vulnerable groups of women whose difficulties accessing services may misleadingly indicate a lower demand. Further research is required to examine this perspective. It is imperative that we draw on the success stories of models which minimise unnecessary barriers to women including the necessity to return to clinic for a post-MTOP pregnancy test. However, to implement such directives leadership and advocacy is required from Australian medical, nursing, midwifery and pharmacy faculties and their associated professional associations. In addition, political will is needed to decriminalise abortion across all states and territories in Australia and ensure dedicated public provision that is based on comprehensive models tailored for all populations.

## Abbreviations

GP: General practitioner
HIC: High income countries
ICP: Integrated care pathway
LMIC: Low and middle income countries
MTOP: Medical termination of pregnancy
MVA: Manual vacuum aspiration
NZ: New Zealand
RANZCOG: Royal Australian and New Zealand College of Obstetricians and Gynaecologists
RCOG: Royal College of obstetricians and gynaecologists
STI: Sexually transmitted disease
STOP: Surgical termination of pregnancy
UK: United Kingdom
US: United States
USAID: United States of America Aid Agency
WHO: World Health Organization
